# From Yeast to Biotechnology

**DOI:** 10.3390/bioengineering9120751

**Published:** 2022-12-02

**Authors:** Alok Patel, Ulrika Rova, Paul Christakopoulos, Leonidas Matsakas

**Affiliations:** Biochemical Process Engineering, Division of Chemical Engineering, Department of Civil, Environmental, and Natural Resources Engineering, Luleå University of Technology, SE-971 87 Luleå, Sweden

Yeasts are widely used in various sectors of biotechnology, from white (industrial) to red (medical). For the development of a strong bioeconomy, cost-effective microbial conversion techniques of renewable and sustainable feedstock into value-added compounds is crucial. Yeasts are a group of microorganisms that have been used for many years to produce fermented food and beverages [1]. Baker’s yeast, *Saccharomyces cerevisiae*, has been used in biotechnology for centuries; its genome was decoded in 1996, paving the way for a comprehensive understanding of its genetics and opening the possibility to improve the performance of the yeast. Aside from its ease of handling and GRAS classification, the discovery of the extremely efficient homologous recombination mechanism is key to considering *S. cerevisiae* as a model organism for genetic transformation [2]. Not only this yeast, but also other non-conventional yeasts, offer properties for bio-process applications such as tolerance for extreme environmental conditions and the capacity to use complex carbon sources in fermentation experiments, which make biotechnological processes effective. However, for the effective outcome of non-conventional yeasts in biotechnology, a number of obstacles must be overcome, such as the production of alternative carbon sources obtained from renewable sources, the development of tailored yeast using the genetic engineering approach, and the optimization of fermentation parameters.

Isoprenoids are one of the most diverse categories of natural compounds and are present in all living organisms. Microorganisms are an appealing option for isoprenoids synthesis despite the fact that they do not accumulate as much isoprenoids as plants do. However, there is a special interest to enhance farnesyl diphosphate (FPP)-derived isoprenoids quantities in microbes due to their high economic value and susceptibility to metabolic engineering. Bröker et al., 2020, suggested a novel route to enhance its quantity by increasing FPP pool in the mevalonate pathway (MVA pathway) of the heterologous overexpression of *tHMGR* and *ERG*13 in host *S. cerevisiae* using the CRISPR/Cas9 system [3]. Furthermore, deletion of the MVA-pathway regulator *ROX*1 resulted in an increase in squalene content in host *S. cerevisiae.* Squalene is an excellent antioxidant and is used in vaccine as adjuvant, which is currently extracted mainly from shark liver oil, posing a risk for shark populations as the overexploitation of sharks could eventually result in their extinction [4]. In this regard, the study conducted by Bröker et al., 2020, is valuable for the microbial over production of squalene, including other FPP-derived isoprenoids [3]. 

Lycopene (C_40_H_56_) is a tetraterpenoid non-provitamin derived from the native mevalonate (MVA) pathway, which gives tomato its red color. Zang et al. (2021) applied Synthetic Chromosome Rearrangement and Modification with the LoxP-mediated Evolution (SCRaMbLE) system for heterologous lycopene biosynthesis in the model yeast *S. cerevisiae* strain YSy 200, which resulted in a final yield of 41.47 mg/L of lycopene. This was a 129.5-fold improvement compared to those obtained with the parent strain [5].

Optogenetics, or the use of light to regulate biological functions, is a flexible approach for stimulating the production of industrial microbes [6]. Various optogenetic methods have been developed to use light as an inducer in real time dynamic control in the production of metabolites from microorganisms. In addition, light is thought to be relatively non-invasive to cells, less expensive, and easier to administer and withdraw from bioreactors than chemical inducers due the numerous medium changes needed [6]. Light is emerging as a potential inducer for bioproduction as metabolic engineering increasingly relies on the fine tuning of complex synthetic pathways.

Single cell oil (SCO) produced by microorganisms such as yeast, bacteria, fungi, and algae, is a suitable feedstock for biodiesel due to its similar fatty acid profile to vegetable oils. Oleaginous yeasts among other microorganisms have tremendous capacities to produce more lipids than those reported with oil producing crops. Some popular species of oleaginous yeasts such as *Rhodotorula toruloides*, *Yarrowia lipolytica*, *Lipomyces starkeyi* and *Cryptococcus curvatus* can synthesize more than 60% of lipids in their cellular compartment. A detailed understanding of the reactions that take place in the metabolic pathway of triacylglycerol synthesis in the oleaginous yeast is available; however, full control of TAG biosynthesis has yet to be still explored. The transcriptional regulatory mechanisms that underpin the oil accumulation system in oleaginous yeasts must be clarified in order to advance our understanding and regulate this system. One way to learn more about transcriptional regulation is the analysis of gene regulatory networks. Aburatani et al. (2020) developed a method based on structural equation modeling (SEM) to understand the regulatory system for the biosynthesis of lipids in oleaginous yeast *Lipomyces starkeyi* [7]. They classified eighty-nine genes related to TAG synthesis into nine groups along with two separate regulatory models for lipid and TAG synthesis [7]. 

Another role of yeast is in the production of valuable compounds of biopharmaceutical interest because of their scalable fermentation, shorter generation time, and eukaryotic type post-translational modifications (PTMs). Recombinant proteins such as insulin, human hemoglobin, and parathyroid hormone are important biopharmaceuticals products produced by yeast. Yeast is a workhorse for the production of vaccines due to expression of different forms of antigens that can be scalable with reasonable cost. Among several yeast species, *Pichia pastoris* is considered one of the most promising options for heterologous protein production in vaccine development [8]. *P. pastoris* synthesizes high cell densities under a controlled environment and the cell lines are genetically stable in terms of the post translational modifications of protein in the endoplasmic reticulum (ER) and resistance to contamination. The efficient recovery of secreted products in downstream processing and limited steps of purification make it cost effective since product loss has a substantial impact on overall product yield. It has already been reported that more than 500 heterologous proteins have been successfully expressed using this host, with several of them being approved for human and commercial use. Human insulin (SuperMan5) and recombinant hepatitis C (HCV) are good examples of recombinant protein expressed in *P. pastoris* [8].

Recalcitrant wastes including toxicants, polymers, and organic pollutants are synthetic substances that are non-biodegradable in nature. Their main origin is human activities, and the water that these pollutants are in is known as refractory wastewater. The REACH (Registration, Evaluation, Authorization and Restriction of Chemicals) law was put into effect by the European Union in 2006 with the goal of protecting human health and the environment. Its application aims to produce additional information on the toxicity and safety of chemicals and prevent the use of compounds that are highly concerning without authorization. However, in order to stimulate growth, metabolic activity, and the production of products when growing yeast, certain chemicals are needed. Pekarsky et al. (2020) suggested that boric acid and cobalt dichloride are harmful chemicals but they can be used to support the cultivation of yeast [9]. They cultivated *Komagataella phaffii* (*P. pastoris*) in the presence of boric acid and cobalt dichloride to produce recombinant proteins. The results suggested that the depletion of boric acid and cobalt from the cultivation media support the growth productivity of this yeast; however, the final recombinant protein quality was affected in various ways by these two chemicals [9]. 

Wine culture has been popular around the globe for many years. By using genetic engineering in *S. cerevisiae,* new frontiers emerge for developing fresh, enhanced, or altered wine characteristic flavors, fragrances, or production processes to meet the demands of an increasingly complex market worth approximately EUR 31.4 billion per year [10]. 

In conclusion, yeasts are thought to be the earliest microbes utilized by humans to ferment food and alcohol. The expertise derived from these old techniques has served as the foundation for current industrial biotechnology. Combining the capabilities of genomic information, metabolic engineering, systems and synthetic biology facilitates the synthesis of a wide range of important primary and secondary metabolism products, and biopharmaceutical recombinant proteins for vaccines.
